# Feasibility of conducting a randomized, placebo-controlled study assessing whether omega-3 fatty acids prevent gout flares when starting urate-lowering treatment

**DOI:** 10.1093/rap/rkac086

**Published:** 2022-10-25

**Authors:** Abhishek Abhishek, Amy Fuller, Georgina Nakafero, Weiya Zhang, Jennifer Dumbleton, Christopher Hawkey, Carol Coupland, Robert Terkeltaub, Michael Doherty

**Affiliations:** Academic Rheumatology, The University of Nottingham, Nottingham, UK; Academic Rheumatology, The University of Nottingham, Nottingham, UK; Academic Rheumatology, The University of Nottingham, Nottingham, UK; Academic Rheumatology, The University of Nottingham, Nottingham, UK; Nottingham Digestive Diseases Centre, The University of Nottingham, Nottingham, UK; Nottingham Digestive Diseases Centre, The University of Nottingham, Nottingham, UK; Division of Primary Care, The University of Nottingham, Nottingham, UK; Veterans Affairs San Diego Healthcare System, San Diego, CA, USA; Department of Medicine, University of California, San Diego, CA, USA; Academic Rheumatology, The University of Nottingham, Nottingham, UK

**Keywords:** gout flare, urate-lowering treatment, omega-3 fatty acids, treat-to-target, feasibility randomized controlled trial

## Abstract

**Objective:**

The aim was to test the feasibility of a randomized controlled trial exploring whether omega-3 fatty acid supplementation limits gout flares during treat-to-target urate-lowering treatment (T2T-ULT).

**Methods:**

Adults with at least one gout flare in the past 12 months and serum urate (SU) ≥360 μmol/l were recruited from general practices (primary method) and randomly assigned 1:1 to receive omega-3 fatty acid supplementation (4 g/day) or placebo for 28 weeks. At week 5, participants began T2T-ULT. The primary outcome was drop-out rate. Secondary outcomes were recruitment rate, outcome data completeness, the number, severity and duration of gout flares between weeks 5 and 28, and study drug compliance.

**Results:**

Ninety-five per cent of randomized participants (*n* = 60) completed all study visits. The primary method recruitment rate was 2.2%. Fifty and 42 participants achieved SU < 360 and 300 μmol/l (6 and 5 mg/dl), respectively. The number of gout flares [median (interquartile range): active 1 (0–2) and placebo 1 (0–2)], flare duration [mean (s.d.): active 7.00 (4.52) days and placebo 7.06 (8.14) days] and time to first flare [hazard ratio (95% CI) 0.97 (0.50, 1.86)] were comparable between both arms. Study drug compliance was high and comparable in both arms [median (interquartile range) returned capsule count: active 57 (26–100) and placebo 58 (27–154)]; red blood cell omega-3 fatty acid index increased twofold in the active arm and remained unchanged in the control arm.

**Conclusion:**

The study demonstrated feasibility and provided useful metrics for conducting a community-based gout flare prophylaxis trial.

**Study registration:**

ISRCTN; https://www.isrctn.com/; ISRCTN79392964.

Key messages

- Preclinical and human observational studies indicate that omega-3 fatty acids have potential to prevent gout flares.
- A trial evaluating omega-3 fatty acid for flare prophylaxis is feasible, although there was no efficacy signal.
- This study provides useful metrics for trials examining the impact of diet and supplementation on arthritic flares.

## Introduction

Gout is effectively managed with long-term treat-to-target (T2T) urate-lowering treatment (ULT) [1, 2]. However, a drawback is an early increase in gout flares, thought to be attributable to remodelling of articular urate crystal deposits. Hence, a limited term of gout flare prophylaxis is recommended in this clinical scenario [3–5]. Unfortunately, co-morbidities and potential drug interactions are highly prevalent in gout patients, which contraindicates one or more of the drugs used for flare prophylaxis (colchicine, NSAIDs and, in rare cases, CSs). As such, there is an unmet need for more safe and effective therapeutic options for gout flare prophylaxis.

Dietary excesses in purines from animal sources and excessive alcohol intake are well recognized as being associated with increased gout flares [6, 7]. Conversely, omega-3 fatty acids [eicosapentaenoic acid (EPA; 20:5n-3) and docosahexaenoic acid (DHA; 22:6n-3)] have the potential capacity to prevent gout flares via inhibition of NLRP3 inflammasome assembly, activation of certain Toll-like receptors, and neutrophil chemotaxis [8–12]. Proof of concept has been demonstrated in a mouse gout flare model [8]. Moreover, in preliminary human studies, omega-3 fatty acids (≥2.4 g/day for ≥4 weeks) reduced circulating cytokine concentrations [13–15], and plasma omega-3 fatty acid index were inversely associated with self-reported gout flares [16].

The aim of this study was to test the feasibility of conducting a randomized controlled trial (RCT) testing the hypothesis that omega-3 fatty acid supplementation limits the incidence, frequency, pain severity and duration of gout flares. Our principal objectives were to assess the drop-out rate (primary outcome), recruitment rate, quality of data collected during gout flare, signal of efficacy, compliance with study drugs and adequacy of blinding, and to decide the primary outcome measure, sample size and number of sites for a future phase 3 RCT.

## Methods

### Study design

This was a single-centre, 1:1 randomized (stratified for contraindication to allopurinol), double‐blind, placebo‐controlled, parallel‐group feasibility study.

### Inclusion criteria

Inclusion criteria were as follows: age ≥18 years; meeting the ACR/EULAR gout classification criteria [17]; self-report of at least one gout flare in the preceding 12 months; serum urate (SU) ≥360μmol/l; willingness to commence T2T-ULT; and on stable analgesics for ≥4 weeks.

### Exclusion criteria

Exclusion criteria were as follows: autoimmune rheumatic disease; Lyme disease; psoriasis; asthma or IBD treated with oral immune-suppressing treatment(s); solid organ cancer; dementia; unable to discontinue NSAIDs, oral CSs, omega-3 fatty acids or colchicine; treated with anticoagulants; exposure to systemic CSs in the last month; allergy to omega-3 fatty acids, fish, gelatine, olive oil, soya and unable to take beef products; pregnant/breastfeeding or planning to do so; use of any unlicensed drug within 3 months before screening or within five half-lives of the investigational agent, whichever was longer; haematological or biochemical abnormality, defined as haemoglobin <85 g/l, white blood cells <3.5 × 10^9^/l, neutrophils <1.5 × 10^9^/l, platelets <100 × 10^9^/l, alanine transaminase >1.5 × upper limit of normal and serum creatinine >2 × upper limit of normal.

### Setting

The study took place in secondary care at Academic Rheumatology, City Hospital Nottingham, Nottingham, UK, between March 2019 and July 2020.

### Recruitment

Staff at general practice surgeries in and around Nottinghamshire generated a list of patients with gout by searching their electronic medical records for diagnosis and previous ULT prescriptions. Next, the staff sent to potentially eligible study participants an information pack about the study, a brief questionnaire about gout, and a pre-paid envelope addressed to the research team. Participants who returned the completed questionnaire and were interested in the study were sent a participant information sheet, reply slip and a pre-paid self-addressed envelope by the research team. In addition, an advert was placed in a local newspaper, and posters were displayed in the outpatient clinics of the Nottingham University Hospital NHS Trust. Potentially interested participants from these sources were invited to contact the research team directly.

### Ethical approval

The study was approved by the Nottingham NHS Research Ethics Committee (IRAS 240464) and Medicines and Healthcare products Regulatory Agency (MHRA) (Eudra CT number 2018-000963-99), registered with International Standard Randomised Controlled Trial Number (ISRCTN) (reference number 79392964) and complied with the Declaration of Helsinki.

### Screening visit

Interested participants were screened against eligibility criteria in a telephone call. Potentially eligible participants were invited for a face-to-face visit, at which point written informed consent was obtained, presence of tophi ascertained and blood collected. Subsequently, eligible participants were invited for a baseline research assessment and randomization visit.

### Randomization

Participants were randomized via an online interface using randomly permuted block sizes of two and four, stratified by contraindication to allopurinol. The randomization sequence was generated by sealed envelope and provided to the independent Clinical Trials Pharmacy, who packaged the study drugs blinded to the investigators. Recruitment was done by trial staff who were not involved in generation of the allocation sequence.

Treatment allocation was concealed from the chief investigator, participants, blinded research outcome assessor and the data analyst for the duration of the trial. Those who were unblinded (e.g. Clinical Trials Pharmacy) did not have any contact with study participants. Study drugs were dispensed by the Clinical Trials Pharmacy in identical, opaque, sealed bottles in the order of the randomization schedule. The study medicine was an unmarked oblong translucent capsule containing pale yellow oil. The placebo capsules produced by Catalent were matched for size, shape, colour, appearance and weight.

### Intervention

Participants were prescribed either the investigational medicinal product (IMP) or matching placebo for 28 weeks. Commercially available unmarked pharmaceutical-grade gelatine-coated soft-gel capsules with 840 mg omega-3 fatty acid ethyl esters in each 1 g capsule (DHA: EPA 380:460) were used as the IMP. Placebo capsules were matching soft-gel capsules containing 1 g pharmaceutical-grade olive oil, manufactured by Catalent (Germany). Participants were instructed to take two capsules twice daily with meals. The dose of IMP or placebo could be reduced if the participant developed intolerance to the prescribed dose.

Participants were commenced on T2T-ULT in week 5, as per the British Society for Rheumatology guidelines [5]. Dose up-titration visits occurred at 2- to 3-weekly intervals. Participants already on ULT at study entry had their dose optimized aiming for SU <300 μmol/l. The T2T-ULT was delivered by an allied health-care professional using principles used in the Nottingham nurse-led care of gout study [1], with the prescriptions signed by a consultant rheumatologist (A.A.).

Gout flares were treated with prednisolone (enteric coated) 30 mg/day with a proton pump inhibitor for ≤1 week. If CSs were contraindicated or if participants preferred not to take CSs, they were prescribed naproxen 500 mg twice a day with a proton pump inhibitor for ≤1 week. Participants were allowed to take analgesics as required during the gout flare.

### Assessments

#### Baseline visit

Participants self-reported their age at first gout flare, number of gout flares in the previous 12 months, medications, co-morbidities, and previous side-effects to ULT; they underwent targeted musculoskeletal assessment for the presence and size of tophi [18] and had height, weight and blood pressure measured. The gout activity questionnaire version 2 (GAQ v.2.0) and the short-form 36 version 2 (SF36v.2) questionnaires were completed [19, 20]. Blood was collected for measuring red blood cell omega-3 fatty acid index (Omegaquant).

#### Gout flare diary

During a gout flare, participants completed the flare diary and recorded the joint(s) affected, daily pain score using a 100 mm visual analogue scale, items required to classify the gout flare [21], medications used and the patient global assessment of response to treatment (PtGART) using a five-point Likert scale. Diaries were returned via post or collected at study visits.

#### Dose up-titration visits

Data on the number of gout flares since last visit, changes in concomitant medication, and adverse events (AEs) were collected. Blood was collected for measuring SU. At the halfway point (week 14) unused IMP/placebo was collected and counted. Owing to the coronavirus disease 2019 (COVID-19) pandemic lockdown, face-to-face visits were replaced with telephone appointments between March and July 2020. Blood could not be collected within this period.

#### End-of-study visit (week 28)

The researcher enquired about the number of flares since last visit, medications and co-morbidity; repeated the targeted musculoskeletal assessment for tophi; and re-measured height, weight and blood pressure. The participants were asked to guess their group allocation and completed the GAQ v.2.0 and SF36v.2. Unused IMP/placebo capsules were collected and counted. Blood was collected for measurement of SU and red blood cell omega-3 fatty acid (Omegaquant). During the restrictions on research visits owing to the COVID-19 pandemic, end-of-study visits were completed remotely using telephone calls for the number of flares, medications and co-morbidities. Participants completed GAQ v.2.0 and SF36v.2 as an online questionnaire in Microsoft Forms or via post.

#### Adverse events

Participants were instructed to contact the study team if they experienced any AEs after consenting to ≤4 weeks after the week 28 visit. Data were also collected at dose up-titration and other visits. Data about concomitant medications taken for AEs were collected.

### Outcomes

The primary outcome was the proportion of randomized participants completing the trial, defined as attending the week 28 research assessment visit, either in person or remotely.

Secondary outcomes were the proportion of potential participants approached by the general practitioner who replied to the research team, agreed to a screening visit, met the eligibility criteria, were randomized, and withdrew from the study owing to side-effects to omega-3 fatty acids; completeness of outcome data on daily pain score, items required to classify gout flares [21], and PtGART in the gout flare diary (data for all self-reported gout flares were used for this); and the number of gout flares between weeks 4 and 28, mean pain score, and self-reported duration of gout flares in flares meeting the classification criteria [21]. Gout flares between weeks 1 and 4 were disregarded because the anti-inflammatory effects of omega-3 fatty acids take 4 weeks to appear. Another key secondary outcome was compliance with study drugs, assessed using returned pill count at weeks 14 and 28, and red blood cell omega-3 fatty acid at week 28.

### Sample size

The main outcome of this study was the drop-out rate. A sample size of 60 was estimated to be sufficient to estimate a drop-out rate (primary outcome) of 20% or higher to within a 95% CI of ±10%. The other purposes of this study were to calculate the sample size of the main trial and to test for a signal of efficacy of omega-3 fatty acids. Such a study does not require a formal sample size calculation, but the sample size for this pilot trial was justified based on published rules of thumb and expected effect size. Several rules of thumb exist and suggest that a two-arm pilot trial should have between 24 and 70 participants to allow calculation of the sample size of a definitive trial [22–25]. However, the effect size of omega-3 fatty acids for preventing flares of gout is not known. Thus, we proposed the inclusion of 60 participants (allowing for ∼20% drop-out rate) in this study to allow us to estimate the pooled s.d. with a reasonable degree of certainty, even if the effect size is 0.2 [25].

### Statistical analyses

Data were analysed using intention-to-treat (ITT) principles. The mean (s.d.), median [interquartile range (IQR)] and *n* (%) were used for descriptive purposes and to assess the suitability of randomization. The distribution of the number of gout flares between weeks 5 and 28 was examined to identify the best-suited regression model to be used in the main trial. Given the small sample size and safety of the investigational medical product, interim analyses were not planned. The likelihood of gout flare in the two arms was compared using Cox regression. Statistical significance was set at *P* < 0.05. Data analyses were performed using STATA MP 16 (StataCorpLLC).

## Results

**Figure 1.**
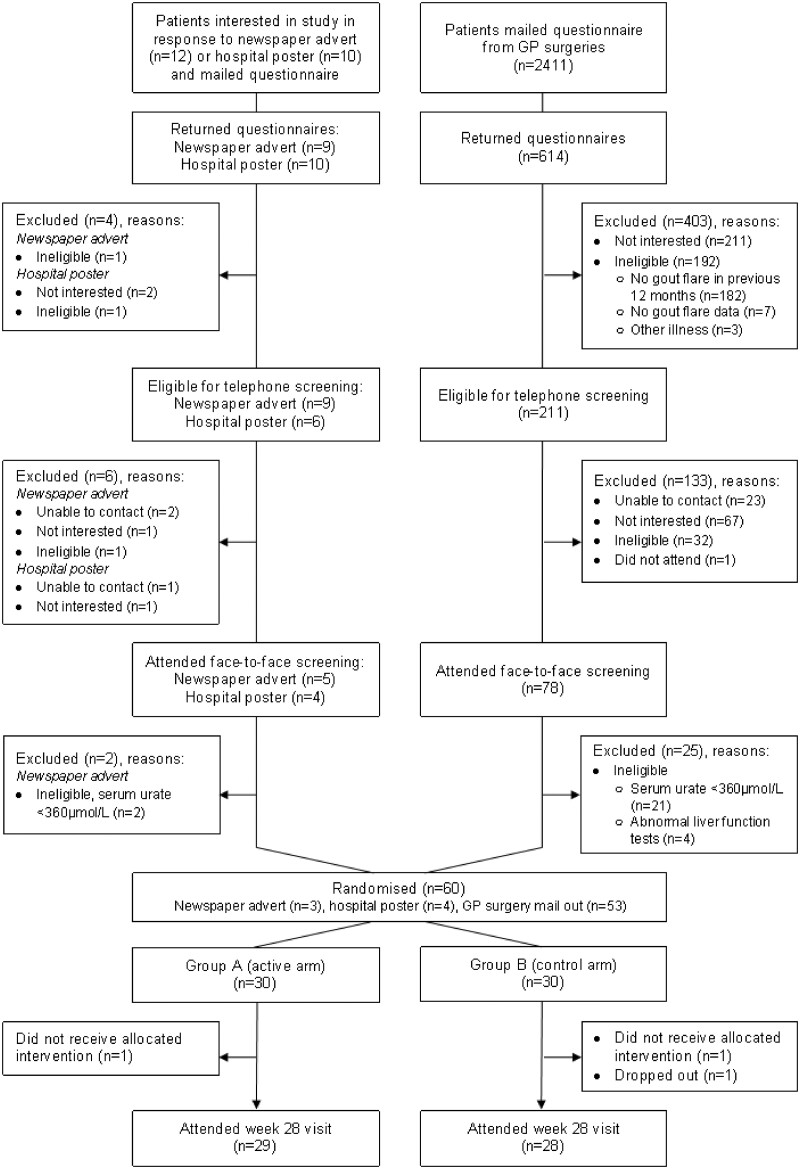
Recruitment flow chart

Of 2411 questionnaires sent to patients by 21 general practices, 614 (25.5%) were returned (Fig. 1). Among them, 211 were eligible and willing to be contacted further. Of these, 78 were screened and 53 randomly assigned to a treatment group. Of the nine people who responded to the advertisement, seven were screened and randomized. Thus, the recruitment rate from the primary route of recruitment was 2.2% (53 of 2411). One participant in each arm did not receive the allocated intervention owing to withdrawal of consent (*n* = 1) or ineligibility (*n* = 1). Participant characteristics were comparable between intervention and control arms at baseline (Table 1).

**Table 1.** Baseline characteristics

Characteristic	Placebo (*n* = 30)	Omega-3 (*n* = 30)
Age, years	58.07 (11.42)	54.93 (12.54)
Sex, male, *n* (%)	28 (93.33)	30 (100)
BMI, kg/m^2^	31.96 (4.98)	30.05 (4.79)
Mean blood pressure, mmHg	107.01 (11.76)	109.03 (11.64)
Diabetes, *n* (%)	1 (3.33)	2 (6.67)
Hypertension, *n* (%)	8 (26.67)	5 (16.67)
Hypercholesterolaemia, *n* (%)	7 (23.3)	4 (13.33)
Ischaemic heart disease, *n* (%)	3 (10)	0
Cerebrovascular disease, *n* (%)	0	1 (3.33)
Age at gout onset, years	47.43 (13.05)	43.28 (13.05)
Number of gout flares in 12 months, median (interquartile range)	2 (1–5)	3 (1–5)
Allopurinol contraindicated, *n* (%)	2 (6.67)	1 (3.33)
Currently on urate-lowering treatment, *n* (%)	13 (43.33)	8 (26.67)
Mean allopurinol dose, mg/day	175 (86.60)	262.5 (91.61)
Serum urate, μmol/l	452.1 (56.15)	454.67 (66.57)
Serum creatinine, μmol/l	86.93 (19.44)	85.67 (24.44)
Red blood cell omega-3 fatty acid index	0.06 (0.01)	0.06 (0.02)
Tophus present, *n* (%)	4 (13.33)	2 (6.67)
Largest tophus size^a^, mm, median (range)	29.5 (18.5–29.5)	33.5 (9–58)
Gout Impact Scale domains		
Gout concern overall	75.21 (23.47)	66.59 (29.47)
Gout medication side-effects	60.42 (26.89)	54.31 (27.20)
Unmet gout treatment need	45.69 (20)	50.30 (19.18)
Well-being during attack	46.67 (24.03)	40.6 (23.14)
Gout concern during attack	51.04 (24.24)	43.53 (23.35)

The mean (s.d.) is reported for continuous variables and *n* (%) for categorical variables.

Median (interquartile range) is reported for number of gout flares and median (range) is reported for largest tophus size.

a Longest dimension of the largest tophus.

Ninety-five per cent (57 of 60) of randomized participants completed all study visits. Additionally, 57 of the 58 participants who received the allocated intervention completed all study visits. One participant in the control arm dropped out, reporting side-effects on increasing the dose of ULT. There was a reduction in SU in both arms (Table 2).

**Table 2.** End of study characteristics

Characteristic	Placebo (*n* = 28)	Omega-3 (*n* = 29)
Number withdrawn	2	1
Number of gout flares 4 weeks post-randomization, median (interquartile range)	1 (0–2)	1 (0–2)
Duration of self-reported gout flare, mean (s.d.), days	7.06 (8.14)	7.00 (4.52)
Urate-lowering treatment^a^		
Allopurinol, *n*	24	28
Allopurinol dose, mg/day	383.33 (137.26)	357.14 (119.96)
Febuxostat, *n*	3	1
Febuxostat dose, mg/day	93.33 (23.09)	80
Benzbromarone, *n*	0	1
Benzbromarone dose, mg/day	0	50
Serum urate^b^, μmol/l	297.14 (51.31)	292.31 (52.88)
Serum urate ≤300 μmol/l	21	21
Serum urate 301–359 μmol/l	4	4
Serum urate ≥360 μmol/l	4	4
Red blood cell omega-3 fatty acid index^c^	0.05 (0.02)	0.10 (0.02)
Number of unused capsules returned, median (interquartile range)	58 (27–154)	57 (26–100)
Participant guess allocation		
Correct, *n* (%)	14 (50)	18 (62)
Could not guess or incorrect, *n* (%)	14 (50)	11 (38)
Tophus present, *n* (%)	2 (7.14)	2 (6.90)
Largest tophus size, mm, median (range)	25.5 (17–34)	33.5 (33–34)
Gout Impact Scale domains		
Gout concern overall	53.13 (24.09)	44.18 (27.55)
Gout medication side-effects	51.34 (28.12)	47.32 (29.14)
Unmet gout treatment need	29.32 (14.87)	25.30 (20.35)
Well-being during attack	50.49 (21.98)	39.90 (22.30)
Gout concern during attack	50.46 (22.46)	41.52 (20.92)

Mean (s.d.) is reported for continuous variables and *n* (%) for categorical variables.

Median (interquartile range) is reported for number of gout flares and median (range) is reported for largest tophus size.

a One person stopped urate-lowering treatment.

b Using last observation carried forward because 29 participants did not have a week 28 visit owing to the coronavirus disease 2019 pandemic.

c Using data from 28 participants who attended for the week 28 visit.

### Quality of data collected during gout flare

Eighty-six gout flares were self-reported in the study period. Of these, data for swollen joints and warm joints were provided for 83 flares (96.5%). The 86 gout flares lasted for a total of 665 days. Data for self-reported daily pain score and PtGART were available for 560 days (84%) and 69 (80.2%) flares, respectively. Data for drugs used to treat gout flares were incomplete in gout flare diaries. None of the diaries had data on all of the following: drug name(s), doses or duration of treatment.

### Signal of efficacy

**Figure 2.**
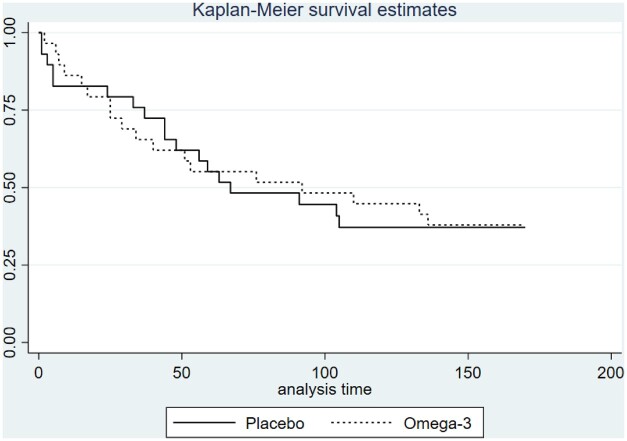
Time to first gout flares stratified by arm

The number of gout flares between week 4 and 28 of randomization was comparable between the two arms (Table 2) and had a negative binomial distribution (Supplementary Fig. S1, available at *Rheumatology Advances in Practice* online). Likewise, the time to first gout flare was comparable between the two arms (Fig. 2), with a hazard ratio (95% CI) of 0.97 (0.50, 1.86). The average duration of gout flares was comparable in the two arms (Table 2). The mean daily pain score was not compared between the two arms because there was 16% of missing data, and multiple imputation was not planned for this feasibility study.

### Compliance with study drugs

Each participant was dispensed 800 capsules of IMP or matching placebo. The compliance with study drugs was high and comparable in both arms (Table 2). The red blood cell omega-3 fatty acid index remained stable in the control arm but increased twofold in the active arm compared with the baseline measurement.

The proportion of participants that correctly guessed their allocated arm was comparable in the two arms (*P* = 0.52, χ^2^ test). The intervention was well tolerated, with one unrelated serious AE in the control arm, and 15 and 11 AEs in the control and active arms, respectively (Table 3). Gastrointestinal AEs were the most common AE.

**Table 3.** Incidence of serious adverse events and adverse events per participant

Event	Placebo (*n* = 30)	Omega-3 (*n* = 30)
Serious adverse event		
Infection [gastroenteritis (viral)]	1	0
Adverse event (organ system)		
Gastrointestinal	3	5
Oral	0	1
Infection (viral)	1	1
Eye	0	2
ENT	2	0
Genitourinary	2	0
Injury/accident	2	1
Respiratory	1	0
Nervous system	2	0
Musculoskeletal	2	1

### Impact of COVID-19

Owing to the COVID-19 pandemic, face-to-face research visits were stopped by the UK government on 23 March 2020. By this time, 28 participants had completed their week 28 visits, with 29 participants still in the trial, who were maintained on the stable doses of ULT and study drugs until their week 28 visit. Blood tests for SU levels and ULT dose up-titration could not be undertaken for 10 participants who had not hit their target SU by their last face-to-face visit. Likewise, end-of-study assessments requiring face-to-face contact (e.g. measurement of size of tophi and collection of blood for SU) could not be completed. The latest available data for SU was imputed as the end-of-study value.

## Discussion

In this study, we examined the metrics of conducting a large phase 3 RCT evaluating the efficacy of omega-3 fatty acids in preventing gout flares when commencing T2T-ULT. We found such a study to be feasible, with a low drop-out rate, good compliance with IMP/placebo, adequate blinding of participants and excellent safety profile of the study drugs. Participants in the control arm had similar omega-3 fatty acid index at baseline, and at the week-28 visit there was a doubling of omega-3 fatty acid index in the intervention arm. Most participants self-reported data that could be used to classify gout flares as meeting the flare criteria of Gaffo *et al.* [21]. However, recruitment was a challenge, because only 2.2% of participants approached by the primary method of recruitment participated in the trial, which was lower than our expectation. This implies that a large number of general practice surgeries are needed to recruit sufficient participants for a definitive study in the context of ULT initiation. There was no evidence for a signal of efficacy for omega-3 fatty acids in preventing gout flares. The number of gout flares, time to the first gout flare and mean duration of each gout flare were comparable in both arms. This dampens the enthusiasm for a future definitive clinical trial for this scenario but does not rule out potential benefit of omega-3 fatty acid supplementation or dietary fish intake on gout flare frequency, as suggested in observational studies [16, 26]. It is possible that reducing flare frequency in the context of ULT initiation might be more difficult to effect than reduction of flares in people already established on ULT or not taking ULT.

The results of this study are generalizable to other interventional gout clinical trials that aim to recruit from primary care gout populations. One limitation of this study was the effects of the COVID-19 pandemic, because we were unable to up-titrate the ULT for many participants to reduce their SU to <300 μmol/l. However, despite this limitation, 70% of participants achieved the treatment target, while 83% achieved the EULAR treatment target of <360 μmol/l. Likewise, <10% of the participants required second- or third-line ULT. This is consistent with previous observations [3, 4, 27].

In this study, we tested the feasibility of a paper diary to collect data during gout flares. The site of gout flare was reported by all, and 96.5% of gout flare diaries had sufficient information to classify the gout flare as meeting the criteria of Gaffo *et al.* [21]. Thus, it is feasible to classify the reported flares as meeting the classification criteria. There was a moderate amount of missing data on PtGART (∼20% of diaries) and daily pain score (16% days). This suggests that adequate participant training is needed when they are used in gout clinical trials.

Strengths of this study include primary care-based recruitment, use of adequate blinding up to data analysis, high adherence to treatment and high retention, with 95% of randomized participants completing the trial. Despite the COVID-19 pandemic, ∼90% of participants achieved the EULAR and ACR SU treatment target [3, 4]. Limitations of the study include the lack of crystal proven diagnosis of gout and the fact the COVID-19 pandemic meant that not all participants were able to escalate ULT to achieve an SU <300µmol/l. There was imbalance in the two arms for prevalence of co-morbidities and ULT. The former is likely to be attributable to the small sample size of the trial, and the latter points to the need to stratify on ULT prescription at baseline during randomization. This study was conducted at a single rheumatology centre with expertise in treating gout to target SU, and despite the COVID-19 pandemic, 88% of participants achieved SU <360 µmol/l with T2T-ULT. Whether similar success in achieving an SU treatment target with T2T-ULT in the context of omega-3 fatty acid or placebo supplementation can be achieved in other centres without a very high level of expertise in managing gout remains unknown and is a limitation of this study. The feasibility of conducting a future multicentre RCT should ideally be evaluated at several research sites in order to develop a broad understanding of potential challenges to the future RCT. Unfortunately, the limited amount of research funding available to us precluded conducting this feasibility study in more than one centre. Nevertheless, the high level of success in the achievement of SU treatment target with T2T-ULT in this study allowed us to evaluate the signal of efficacy for omega-3 fatty acids in gout flare prophylaxis with confidence. This study also provided useful metrics on recruitment, drop-out, adequacy of blinding, and tolerability of the IMP and placebo. However, given that this was a single-centre study, the feasibility of achieving SU treatment target with T2T-ULT, recruitment rate and drop-out rate ought to be monitored in an internal pilot of a future multicentre RCT.

In summary, this study provided useful metrics that might be useful in future gout clinical trials of dietary and diet supplementation measures to limit flares of arthritis. It demonstrated the feasibility of using a paper diary for data collection during gout flares. Omega-3 fatty acid supplements were shown to be well tolerated by gout patients, and adequate blinding was obtained by use of a specific placebo. Although there was no signal of efficacy of omega-3 fatty acid supplementation for limiting gout flares in T2T-ULT initiators, the results of the trial do not rule out testing this or the potential of a diet rich in omega-3 fatty acids or other compounds to limit gout flares in a full RCT.

## Supplementary data

Supplementary data are available at *Rheumatology Advances in Practice* online.

## Supplementary Material

rkac086_Supplementary_Data

## Data Availability

The data underlying this article will be shared on reasonable request to the corresponding author.
