# Supplementary figures and images for: Feasibility of conducting a randomized, placebo-controlled study assessing whether omega-3 fatty acids prevent gout flares when starting urate-lowering treatment
Source: Rheumatol Adv Pract. 2022 Oct 25;6(3):rkac086. doi: 10.1093/rap/rkac086 (PMC9667976; doi:10.1093/rap/rkac086)

**Supplementary Figure S1. Distribution of gout flares stratified by arm**

Placebo


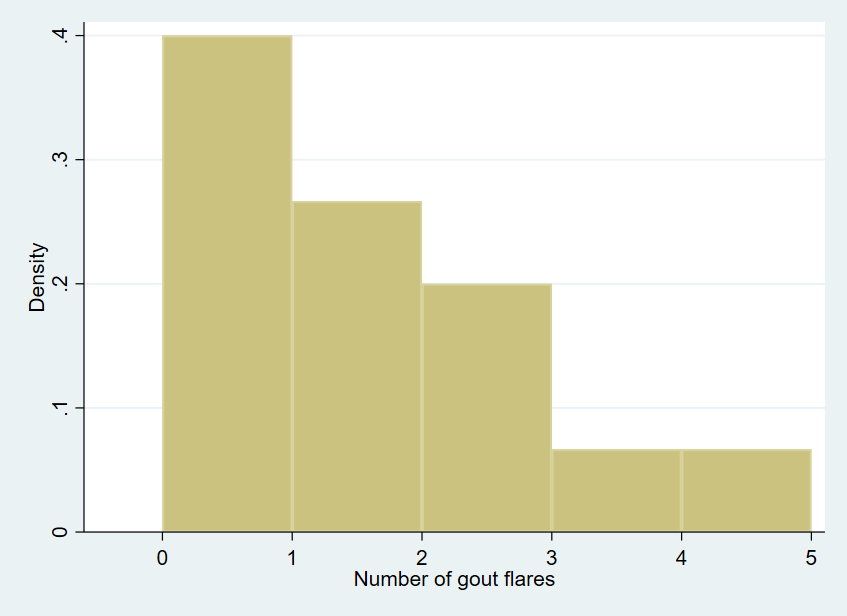


Omega-3


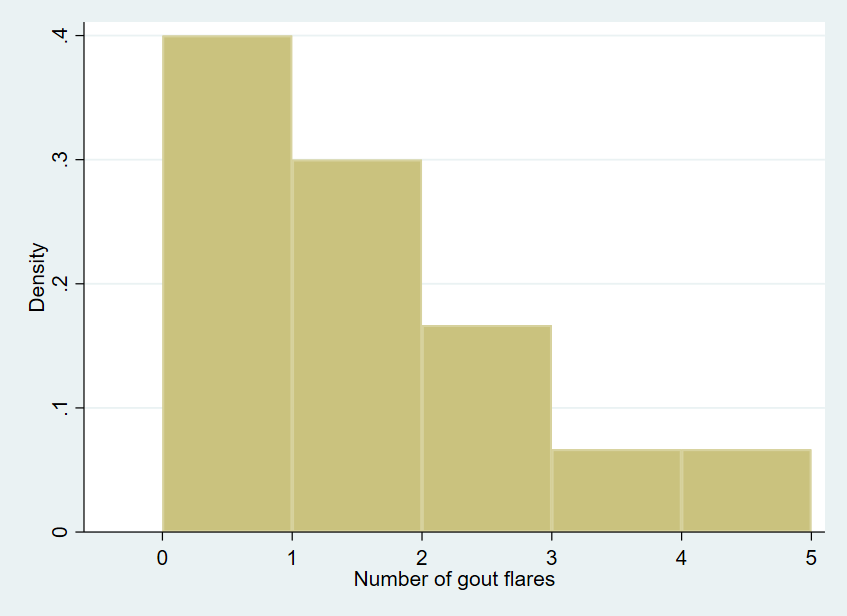

Supplement: rkac086_Supplementary_Data [file rkac086_supplementary_data.docx]
